# Analysis of risk factors for complications in echocardiography-guided percutaneous intramyocardial septal radiofrequency ablation

**DOI:** 10.1186/s13019-024-02934-1

**Published:** 2024-07-16

**Authors:** Hanzhi Wang, Jifang Cheng, Qi Chen, Zhaoxia Pu, Huajun Li

**Affiliations:** Department of Nursing, The Second Affiliated Hospital of Zhejiang University School of Medicine, 88 Jiefang Road, Hangzhou, 310009 China

**Keywords:** Hypertrophic cardiomyopathy, Radiofrequency ablation, Complication, Risk factor

## Abstract

**Background:**

The feasibility of percutaneous intramyocardial septal radiofrequency ablation (PIMSRA) for the treatment of hypertrophic obstructive cardiomyopathy (HOCM) has been previously reported. However, limited investigation has been conducted regarding the complications associated with this procedure.

**Objective:**

This study aims to analyze the risk factors affecting the occurrence of complications during PIMSRA, such as pericardial effusion, ventricular premature beats, and interventricular septal perforation. In this study, the optimal cut-off values for these risk factors are also explored, and corresponding strategies for prevention are proposed.

**Methods:**

A total of 101 patients diagnosed with HOCM who underwent the PIMSRA procedure from 2021 to 2022 were included in this retrospective analysis. Patients were classified into subgroups with or without complications based on procedural records. Univariate and multivariate regression analyses were conducted to identify independent risk factors for complications during the PIMSRA procedure.

**Results:**

There were 48 patients with complications and 53 patients without complications. The heart rate at the start of the procedure and the maximum left ventricular outflow tract gradient (LVOTG) were independent risk factors related to PIMSRA complications. The optimal cut-off values for predicting complication occurrence were a heart rate > 49 bpm at the start of the procedure (OR: 3.79, 95% CI: 1.64–8.78, *p* = 0.002) and a maximum LVOTG > 92 mmHg (OR: 2.57, 95% CI: 1.15–5.75, *p* = 0.022), respectively.

**Conclusions:**

The occurrence of PIMSRA complications is primarily associated with the heart rate at the start of the procedure and the maximum LVOTG. It is recommended to establish a comprehensive control plan to minimize the risk of complications during PIMSRA procedures.

## Introduction

Hypertrophic obstructive cardiomyopathy (HOCM) is a genetic cardiovascular disorder characterized by left ventricular hypertrophy, resulting in left ventricular outflow tract obstruction (LVOTO). HOCM can lead to symptoms such as chest pain, shortness of breath, fatigue, arrhythmias, and in some cases, cardiac death [1]. Currently, HOCM can be treated through septal reduction procedures, implantation of cardioverter-defibrillators, and other methods to improve clinical symptoms, relief LVOTO, and prevent sudden cardiac death [2]. For HOCM patients resistant to drug therapy, invasive interventions play a crucial role in treatment. Septal reduction therapies, such as surgical septal myectomy and alcohol septal ablation (ASA), have demonstrated ongoing improvements in safety and efficacy. Recently, percutaneous intramyocardial septal radiofrequency ablation (PIMSRA) has emerged as a novel and clinically feasible procedure for treating HOCM [3, 4]. The technique utilizes radiofrequency energy to ablate a small area of heart muscle causing obstruction. However, it is crucial to acknowledge the potential occurrence of complications associated with PIMSRA [5]. Common complications during PIMSRA procedures include pericardial effusion, premature ventricular contraction, and myocardial perforation etc.

In this study, our objective is to analyze the risk factors associated with complications during PIMSRA. By exploring these aspects, we intend to provide valuable insights that not only enhance the safety profile of PIMSRA but also refine its application in the context of HOCM management.

## Methods

### Patient population

This was a retrospective, single-center study enrolled HOCM patients who underwent PIMSRA procedure from 2021 January to 2022 October at a tertiary hospital. Inclusion criteria were as follow: (1) HOCM detected by echocardiogram (Septal thickness ≥ 15 mm and resting or provocable LVOTG ≥ 30 mmHg); (2) Patients exhibited significant clinical symptoms; (3) New York Heart Association (NYHA) functional classification was ≥ grade III; (4) Resistive to drug therapy or intolerance to drug side effects. Exclusion criteria included: (1) Concurrent conditions requiring surgery (severe mitral valve organic lesions, coronary artery disease requiring coronary artery bypass grafting); (2) Heart failure (persistent resting heart failure symptoms despite intensified anti-heart failure treatment, left ventricular ejection fraction < 50%); (3) Coagulation abnormalities.

### PIMSRA procedure and complications

During the PIMSRA procedure, patients were positioned in the left lateral decubitus posture following the administration of general anesthesia. Continuous monitoring of heart rate, blood pressure, and blood flow velocity was maintained throughout the intervention. Transthoracic echocardiography (TTE) was performed with the EPIQ 7 C Ultrasound System (Philips Medical Systems) with a S5-1 and X5-1 transducer (1.0 to 5.0 MHz). Measurements of the septal thickness, maximum LVOTG, and left ventricular ejection fraction were obtained according to the recommendations of the American Society of Echocardiography [6]. A temporary pacing wire was inserted through the right internal jugular vein, reaching the apex of the right ventricle. The TTE-guided PIMSRA procedure involved the insertion of a 17G radiofrequency electrode needle (Cool-tip RF Ablation System and Switching Controller, Medtronic Minimally Invasive Therapies, Minneapolis, Minnesota) into the hypertrophied interventricular septum (IVS) via a percutaneous intramyocardial approach. For cases involving hypertrophic anterior and posterior IVS associated with LVOTO, both regions were ablated to ensure efficacy. Successful ablation was verified by assessing contrast perfusion defects in the ablated regions and comparing them with the hypertrophic IVS observed before the procedure. After ablation, patients were transferred to the cardiac intensive care unit for continuous monitoring lasting a minimum of 24 h. Patient classification was based on the presence of post-PIMSRA complications, including pericardial effusion, pericardial tamponade, premature ventricular contractions (PVC), ventricular tachycardia (VT), and ventricular septal defects (VSD).

### Statistical analysis

Continuous variables are expressed as mean ± standard deviation (SD) or median (interquartile range). Categorical variables are presented as numbers and relative frequencies. Baseline clinical characteristics were compared between patients with and without complication. Continuous values were compared by Student’s t test or Mann-Whitney’s test. Categorical variables were compared using Fisher’s exact test or Chi square test. Univariate and multivariate logistic regression methods were used to identify the risk factors associated with occurrence of complications during PIMSRA. For potential risk factors, receiver operating characteristic (ROC) curve analysis was used to determine the optimal cutoff value in predicting complications. The area under the curve (AUC) were also used for assessment. A p-value < 0.05 was considered statistically significant. The statistical analysis was performed using MedCalc (MedCalc Software Inc., Ostend, Belgium).

## Results

A total of 101 patients were enrolled in this study. Among them, 48 patients (47.52%) experienced at least one post-PIMSRA complication. Specifically, 39 patients (38.61%) developed pericardial effusion, 4 patients (3.96%) experienced pericardial tamponade, 3 patients (2.97%) had PVC, 4 patients (3.96%) encountered VT, and 2 patients (1.98%) exhibited VSD. Four patients experienced more than one post-PIMSRA complication.

### Patient characteristics

Table 1 presents baseline and clinical characteristics of the study population. A significant difference in heart rate at the start of the procedure was observed between patients with and without complications (*p* = 0.013), but not for heart rate at the end of the procedure. There were no significant differences in baseline characteristics, including age, BMI, blood pressure, and cardiac risk factors (diabetes, hypertension, and smoking) between the two groups. Additionally, other clinical characteristics, such as left ventricular ejection fraction (LVEF), septal thickness, blood flow velocity, maximum LVOGT, and cardiac troponin (cTn), were comparable between the subgroups.

**Table 1:** Comparison of baseline characteristics between two groups of patients with and without complications

	Complication (*n* = 48)	No complication (*n* = 53)	*p*
Age, y	58.00 (49.25, 67.00)	59.00 (49.25, 67.00)	0.956
BMI, kg/m^2^	24.68 ± 3.78	24.86 ± 2.60	0.784
Systolic blood pressure, mm Hg	127.29 ± 26.74	124.60 ± 17.71	0.550
Diastolic blood pressure, mm Hg	74.38 ± 12.19	70.25 ± 10.64	0.073
LVEF, %	66.20 (63.35, 72.50)	68.00 (62.58, 72.98)	0.516
Heart rate at procedure start, beats/min	56.71 ± 11.73	51.79 ± 10.73	**0.013**
Heart rate at procedure end, beats/min	62.00 ± 17.95	58.17 ± 11.86	0.210
Diabetes	2 (4.17)	3 (5.66)	0.993
Hypertension	20 (41.67)	23 (43.40)	0.989
Smoker	13 (27.08)	10 (18.87)	0.456
Drinker	9 (18.75)	12 (22.64)	0.814
Septal thickness, cm	2.12 (1.70, 2.44)	2.07 (1.66, 2.48)	0.732
Blood flow velocity, m/s	4.46 ± 5.20	3.34 ± 1.38	0.151
Maximum LVOTG, mm Hg	107.50 (79.00, 134.00)	89.00 (72.50, 123.00)	0.122
cTn, ng/mL	0.023 (0.010, 4.450)	0.066 (0.017, 9.990)	0.128

Values are expressed as mean ± standard deviation, median (interquartile range), or number (%). BMI: body mass index; LVEF: left ventricular ejection fraction; LVOTG: left ventricular outflow tract gradient; cTn: cardiac troponin

### Risk factor logistic regression

As indicated in Table 2, the heart rate at the start of procedure emerged as a predictor of post-PIMSRA complications, with an odds ratio (OR) of 1.04 (95% confidence interval [CI] 1.01–1.08, *p* = 0.037) in univariate analysis. In multivariate regression, the heart rate at the start of procedure remained a predictor with an OR of 1.11 (95% CI 1.03–1.19, *p* = 0.004). Additionally, the maximum LVOTG also demonstrated predictive capability for complications, with an OR of 1.01 (95% CI 1.00-1.03, *p* = 0.046).

**Table 2:** Univariable and multivariate logistic regression analysis

	Univariate OR	*p*	Multivariate OR	*p*
Age, y	1.00(0.97–1.03)	0.820	0.99(0.93–1.05)	0.793
BMI, kg/m^2^	0.98(0.87–1.11)	0.782	0.86(0.66–1.10)	0.228
LVEF	0.98(0.92–1.04)	0.572	0.96(0.86–1.08)	0.534
Septal thickness, cm	1.17(0.61–2.27)	0.636	0.96(0.15–6.30)	0.971
Heart rate at procedure start, beats/min	1.04(1.01–1.08)	**0.037**	1.11(1.03–1.19)	**0.004**
Maximum LVOTG, mm Hg	1.01(0.99–1.02)	0.160	1.01(1.00-1.03)	**0.046**
cTn, ng/mL	0.99(0.97–1.02)	0.559	0.97(0.91–1.03)	0.281

95% confidence interval (95% CI) in parentheses. OR: odd ratio; BMI: body mass index; LVEF: left ventricular ejection fraction; LVOTG: left ventricular outflow tract gradient; cTn: cardiac troponin

### Cut-off value in predicting complication

**Fig. 1:**
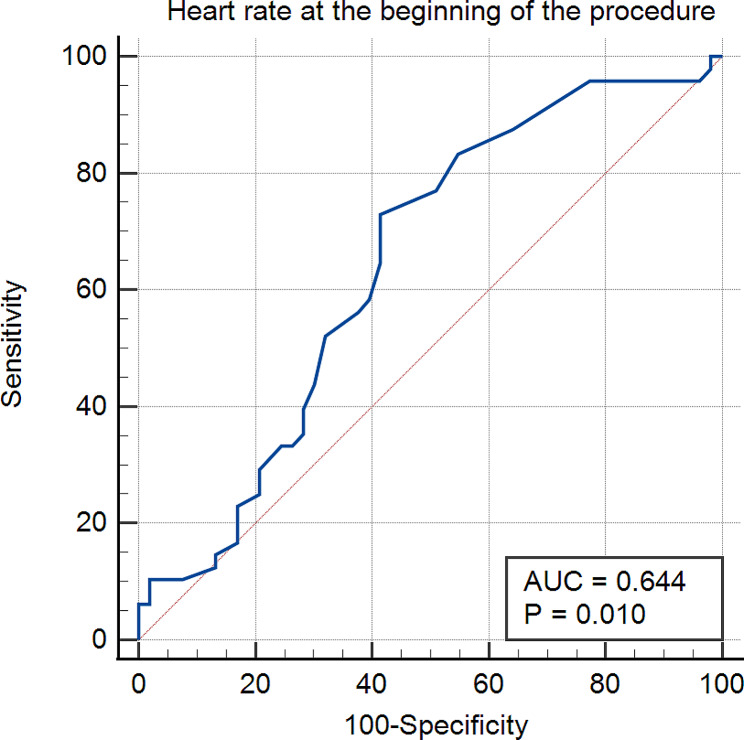
ROC Curve of the heart rate at the beginning of the procedure versus complications in PIMSRA

**Fig. 2:**
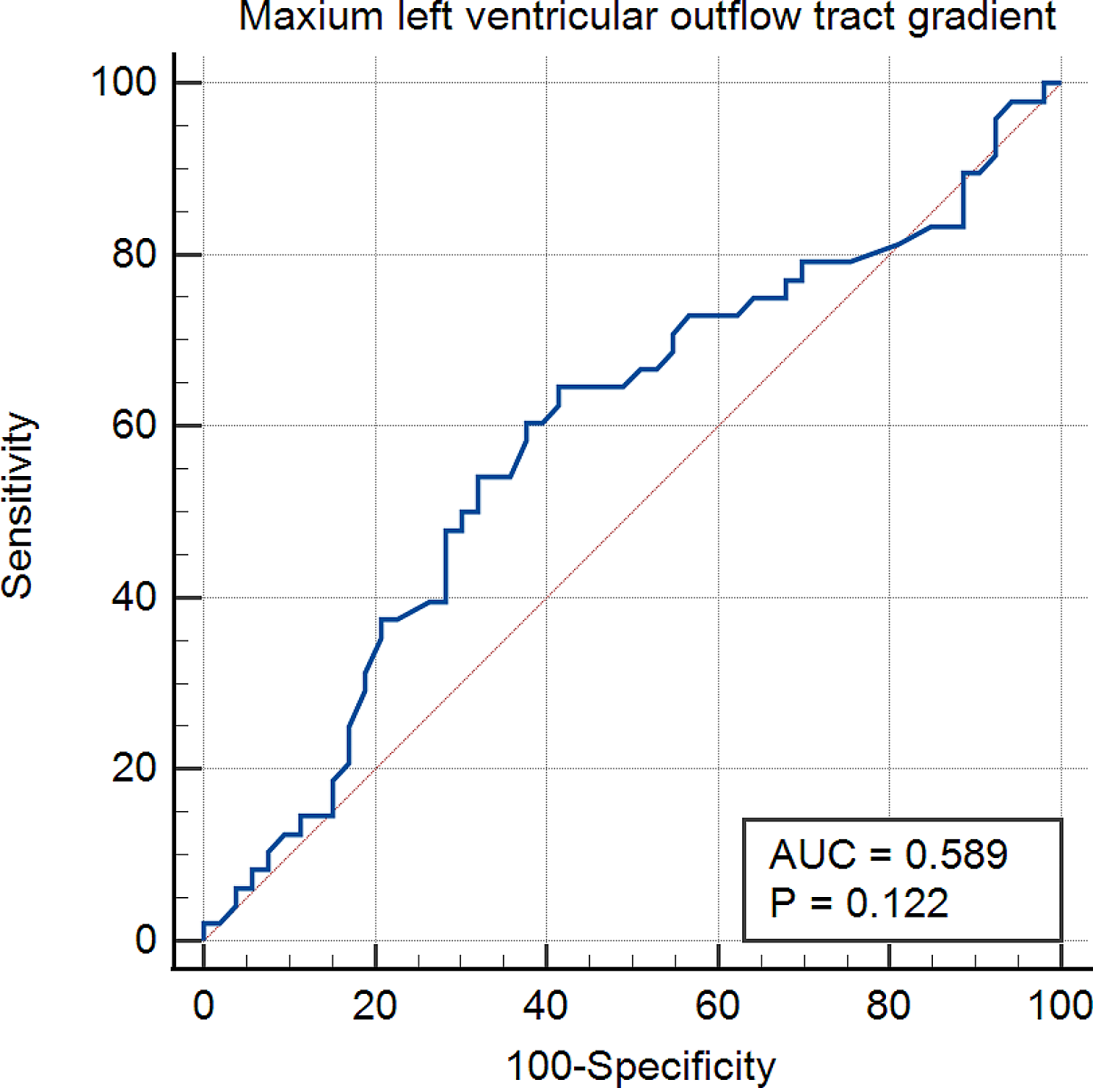
ROC Curve of LVOTG versus complications in PIMSRA

In ROC analysis, the optimal cut-off values for predicting complication occurrence were the heart rate > 49 bpm at the start of the procedure (sensitivity: 72.9%, specificity: 58.5%, AUC = 0.644, *p* = 0.010) (shown in Fig. 1) and the maximum LVOTG > 92 mmHg (sensitivity: 64.6%, specificity: 58.5%, AUC = 0.589, *p* = 0.122) (shown in Fig. 2).

## Discussion

We explored the association between potential risk factors and complications of the PIMSRA procedure. The main findings of this study indicate that, for HOCM patients undergoing the PIMSRA procedure, a heart rate > 49 bpm at the start of the procedure and a maximum LVOTG > 92 mmHg before the procedure are independent predictors of complications.

Hypertrophic cardiomyopathy (HCM) is one of the most common inherited cardiac disorders, manifesting with symptoms such as chest pain, fainting, heart murmur, a sensation of rapid, fluttering, or pounding heartbeats, and shortness of breath. HOCM is a variant occurs in 70% of HCM patients, and can be associated with disabling symptoms and an elevated risk of sudden death [7]. Initial therapy for symptomatic patients involves negative inotropic drugs that diminish contractile force. However, a significant number of patients exhibit an insufficient response to maximal therapy, necessitating more aggressive septal reduction strategies. Current therapies for drug-refractory symptoms include surgical myectomy and ASA. Both procedures effectively improve clinical symptoms and reduce outflow gradients. PIMSRA is a novel procedure that combines the advantages of surgical myectomy and ASA while avoiding sternotomy and minimizing damage to the conduction system distributed underneath the endocardium [8]. Wang et al. demonstrated the clinical applicability of the Liwen Liu RF™ ablation system for RIMSRA in the treatment of drug-resistant HOCM, with all 68 patients achieving satisfactory treatment results [3]. In the 12-month follow-up, the resting LVOTG significantly decreased compared to baseline, and there was a notable reduction in provoked LVOTG during physical exercise. Subsequently, a single-arm, open-label study of PIMSRA in a larger population of patients with drug-refractory HOCM was conducted, revealing that PIMSRA may be an effective procedure for relieving left ventricular outflow tract obstruction and associated symptoms [4].

While the PIMSRA procedure is highly innovative and effective, it remains technically challenging, with potential complications [9]. In this study, we investigated these complications associated with the PIMSRA procedure, including pericardial effusion, pericardial tamponade, PVC, VT, and VSD, as well as the risk factors associated with these complications. It was observed that patients with a heart rate > 49 bpm at the beginning of the procedure faced a higher risk of complications. This heightened risk may be attributed to several factors. High heart rate increase myocardial oxygen consumption and shorten diastolic filling time, leading to an imbalance in myocardial oxygen delivery and reduced ventricular diastolic filling efficiency. The imbalance in myocardial oxygen delivery leads to myocardial hypoxia and necrosis, releasing inflammatory factors that cause pericarditis and ultimately pericardial effusion. Reduced ventricular diastolic filling efficiency can increase pericardial pressure and promote the accumulation of pericardial fluid, resulting in cardiac tamponade. A higher heart rate indicates increased hemodynamic stress, suggesting a less favorable physiological state [10]. Additionally, a higher heart rate can impair the myocardial relaxation phase, reducing the heart’s compliance and potentially affecting the stability of the procedure, thereby increasing the risk of complications. Previous studies have demonstrated risk lies behind higher heart rate. Kurgansky et al. reported that high pulse rate, both at the time of diagnosis and during follow-up, is strongly associated with increased risk of adverse outcomes in in heart failure patients with reduced ejection fraction [11]. Shen et al. found that a higher mean heart rate was independently associated with an increased risk of long-term all-cause mortality in patients with ST-segment elevation myocardial infarction [12]. Therefore, the reduction of heart rate is a clinically meaningful goal. From a nursing perspective, it is essential to verify the dosage, frequency, and timing of patients’ administration of heart rate control medications (such as beta blockers) during handovers. Recording patients’ adherence to medication treatment, along with monitoring their heart rate, occurrence of premature beats, atrioventricular conduction block, and other arrhythmias during hospitalization, is crucial. This will contribute to the development of a more comprehensive and accurate patient-specific heart rate control plan. A maximum LVOTG > 92 mmHg before the procedure is another independent predictor for PIMSRA-related complications. A higher maximum LVOTG before the procedure indicates a more severe obstruction in the left ventricular outflow tract. This pre-existing obstruction could make the PIMSRA procedure technically challenging and increase the likelihood of complications. Moreover, reduced blood pressure, tachycardia, heightened anxiety, and inadequate blood volume due to diarrhea, vomiting, or increased urination can further contribute to an elevated LVOTG, thereby increasing the risk of procedure. Hence, a dialogue between patients and their care team that includes full disclosure of all testing and treatment options, discussion of the risks and benefits of those options would be particularly relevant in the management of HOCM patients and would ultimately benefit the treatment outcome.

This study has several limitations that warrant consideration. First, being a single-center study with a limited patient population, there is a potential for bias, and caution should be exercised in extrapolating the mentioned threshold values to broader populations. Second, as a retrospective study, we cannot directly validate the potential positive benefits of controlling the risk factors mentioned in the study. Moreover, given the relatively small sample size of our current study, it is possible that with an increased sample size, new indicators may emerge with statistically significant associations. Despite these limitations, our study provides valuable insights into the risk factors associated with PIMSRA complications. However, it is essential to interpret these findings within the context of the study’s constraints. Future endeavors should involve larger, multicenter studies to further explore and confirm the identified risk factors, enhancing the clinical relevance and applicability of our results.

## Conclusions

For patients with HOCM undergoing PIMSRA, a heart rate > 49 bpm at the start of the procedure and a maximum LVOTG > 92 mmHg before the procedure are independent predictors of procedure-related complications. In cardiology nursing, it’s crucial to give special attention to patients displaying these characteristics to improve overall clinical outcomes.

## Data Availability

The datasets that support the findings of this study are available from the corresponding author on reasonable request.
